# Assessment of Sensory Processing Characteristics in Children Between 0 and 14 Years of Age: A Systematic Review

**DOI:** 10.22037/ijcn.v15i1.21274

**Published:** 2021

**Authors:** Marjan SHAHBAZI, Navid MIRZAKHANI

**Affiliations:** 1 Occupational Therapy, School of Rehabilitation, Shahid Beheshti University of Medical Sciences, Tehran, Iran; 2 Physiotherapy Research Center and Department of Occupational Therapy, School of Rehabilitation, Shahid Beheshti University of Medical Sciences, Tehran, Iran.

**Keywords:** Sensation, Outcome Assessment, Child

## Abstract

**Objectives:**

processing disorder (SPD) is a neurodevelopmental disorder that can negatively affect objective, emotional, and behavioral functioning. Therefore, assessing sensory processing is critical in children. This study aimed to provide a current comprehensive list of assessment instruments special about sensory processing in children aged between 0 and 14 years.

**Materials & Methods:**

This systematic review focused on pediatric assessment of sensory processing. Three electronic databases (Google Scholar, Web of Science, Scopus, PubMed, and ProQuest) were comprehensively searched for eligible studies, and language restriction (English) was applied.

The search strategy consisted of keywords and medical subordinate headings for sensory processing and various pediatric assessment tools.

**Results:**

Thirty-four assessment tools were identified, of which nine met the predefined inclusion criteria. The test of ideational praxis, clinical observations of proprioception, and pediatric clinical test of sensory interaction for balance were clinical observational assessment tools.

The final tool was a caregiver or teacher reported questionnaire. The obtained studies evaluated the clinical use and psychometric properties of these nine assessment tools.

**Conclusion:**

The result of this study indicated that each of the sensory processing assessment tools considered various aspects of sensory processing.

Selecting the most appropriate assessment tools to measure sensory processing function in children depends on specific components of sensory processing that need to be evaluated.

## Introduction

“Sensory processing is defined as registration, modulation, integration, and organization of sensory inputs to execute successful adaptive responses to situational demands, and in this way, engage meaningfully in daily occupations (1)”. The defect in this process leads to sensory processing disorder (SPD). SPD expresses dysfunctions in the capacity to regulate and organize the degree, intensity, and nature of responses to sensory inputs in a graded and adaptive manner. These disorders have a long-term impact on a child’s life at home, at school, and in the community (2).

Based on clinical experience, the prevalence of SPD has been determined to be 5 to 10 percent for children without disabilities, but 40 to 88 percent for children with various disabilities. Nevertheless, the frequency estimate of SPD based on parent's perception is 5.3 percent in preschool children (3).

Dunn’s model of sensory processing presents behavioral responses to sensations. This model suggests four basic patterns of sensory processing emerging from the interplay of the neurological threshold and self-regulation. The neurological threshold is a personal range of thresholds for noticing and reacting to different sensory events in daily life. People with a low sensory threshold notice and react to stimuli more often because their neurological system activates more easily and responds more readily to sensory events. On the other hand, people with a high sensory threshold often miss stimuli that others notice easily because their neurological system needs stronger stimuli to be activated. Self-regulation is a continuum of a behavioral construct. One end shows those who produce a passive strategy toward sensory events, like remaining at a place with many sensory inputs that makes them feel uncomfortable and respond with disappointment. The other end indicates people that use an active approach; for example, adjusting one’s position to influence a manageable amount of sensory inputs. Accordingly, four patterns can result from the intersection of the neurological threshold and self-regulation; they are (1) registration (represents high neurological thresholds with passive self-regulation), (2) seeking (represents high neurological thresholds as well, but seekers have an active self-regulation strategy and generate new ideas), (3) sensitivity (represents low neurological thresholds and a passive self-regulation strategy, and (4) avoiding (represents low neurological thresholds as well, with an active self-regulation strategy. People with acute responses to a sensory event are likely to have interfered daily life. This model provides assessment and intervention approaches for therapists to promote people’s participation in major domains. Dunn's model refers to individuals at the extremes of the continuum as experiencing atypical sensory processing patterns, while other models refer to these people as undergoing SPD (4).

Functional impairments associated with SPD include decreased social skills, decreased collaboration in daily practice, lack of adaptive responses, impaired self-confidence or self-esteem, diminished fine and gross motor skill development; delay in learning and language, and decreased executive and self-regulatory function. These factors demonstrate why sensory processing is recognized as a domain of concern in the pediatric field (5,6,7).

Based on the results of various studies and significance of factors like negative effect of SPD on children’s functional abilities, evaluation of sensory processing is one of the essential parts of assessment for children with SPD (7,8). This study aimed to provide a current comprehensive list of pediatric assessment tools particularly developed for sensory processing in children between 0 and 14 years of age. This systematic review summarizes the psychometric characteristics of the tools evaluating sensory processing. Based on the result of our review, professionals can use suitable and valid sensory processing assessment tools fundamental to identifying and optimizing sensory processing in SPD patients.

Review question

1. What tools are available for assessing sensory processing in SPD patients?

## Materials & Methods

This study was designed as a review for running overall reported assessment tools for sensory processing in the past 29 years, from 1 January 1990 to January 31, 2019. The study was approved by the Ethics Committee of Shahid Beheshti University of Medical Sciences with the code IR.SBMU.RETECH.REC.1396.1393. 

Search strategy for identifying relevant studies

The third search method was used to identify eligible studies. Initially, we investigated five English databases (PubMed, Scopus, Web of Science, ProQuest, and Google Scholar). Then, we electronically searched a specialized journal (American Journal of Occupational Therapy, physical and occupational therapy in pediatrics and occupational therapy in healthcare). Finally, the reference lists of the collected articles were searched for relevant studies.

 Bibliographic database searches

The search strategy included MeSH databases, and text words included: (“child behavior” OR “sensation” OR “psychomotor performance” OR “sensory processing” OR “perception” OR “sensorial modulation” OR “sensation disorder”) AND (“psychometrics” OR “outcome assessment” OR “questionnaire” OR outcome and process assessment”) AND (“pediatrics” OR “child”). The PubMed search strategy shown in Table 1 was adapted for the other databases.

**Table 1:** The PubMed search strategy

Search	Search terms
1	“Child” OR “Pediatrics “
2	“Sensation” OR “Sensation disorder”
3	“Outcome assessment” OR “Outcome and process assessment”
4	# 1 AND # 2
5	Studies published in English

Study selection

**Fig 1:**
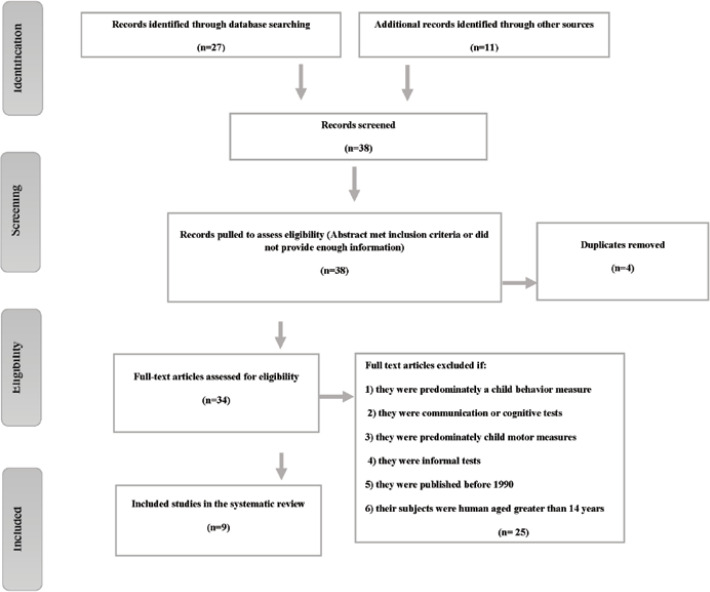
Selection of studies for review of sensory processing assessment tools available in the literature in children between 0 and 14 years of age

A total of 38 articles were identified through the original search process. Based on title and abstract screening, four articles were excluded as they did not meet the inclusion criteria. Of the remaining 34 full-text articles, 25 were excluded because they met the exclusion criteria. The remaining nine articles were selected for review (Figure 1). 

Inclusion criteria 

Articles were reviewed if they met all the following inclusion criteria: (1) being used to assess sensory processing in children; (2) being published in English; (3) being commercially or electronically available (4) being among psychometric studies, and (5) having assessment items mostly related to sensory processing outcomes (visual processing, auditory processing, vestibular processing, proprioceptive processing, smell processing, and tactile processing).

Exclusion criteria

Articles were excluded if they met any of the following exclusion criteria: (1) being predominately a child behavior measure; (2) being a communication or cognitive test; (3) being an informal test; (4) being published before 1990; (5) having subjects with the age greater than 14 years, and (6) having tools with the focus mainly on motor skills.

Bias avoidance

To avoid bias, extraction and quality evaluation of published articles were properly performed by two academic researchers. If the articles were rejected, the reason for their refusal was mentioned and any disagreement between the two authors was solved with discussion.

methodological quality assessment and data report

The methodological quality of the included articles was assessed using the *can child* outcome measure rating form. 

Data extraction 

After excluding articles, the full texts of the remaining articles were carefully studied. Afterward, related studies were selected and irrelevant ones were excluded. A modified version of the *can child* outcome measure rating form was applied to assess the clinical use, reliability, validity, and responsiveness of each included assessment tool. Additional assessment characteristics were extracted and documented including targeted age range, scoring, type of test (criterion or norm-referenced), author(s), year of publication, publisher, description, responders, and number of items.

## Results

In this study, 38 articles were selected and after reviewing their full texts, they were assessed for eligibility. Finally, 25 articles were excluded. Table 2 lists the 25 articles that were excluded based on the inclusion and exclusion criteria.

Only nine assessment tools met the predefined inclusion criteria: (1) the sensory rating scale (10); (2) the sensory processing measure (SPM) (11); (3) the test of ideational praxis (TIP) (12); (4) the sensory experience questionnaire (SEQ) (13); (5) the clinical observation of proprioception (COP) (14); (6) the sensory profile 2 (15); (7) the participation and sensory environment questionnaire (P-SEQ) (16); (8) the pediatric clinical test of sensory interaction for balance (P-CTSIB) (17); and (9) the sensory processing three dimension scale (18). Table 3 provides a summary of the characteristics of these tools.

**Table 2:** Assessments excluded and their corresponding exclusion criteria

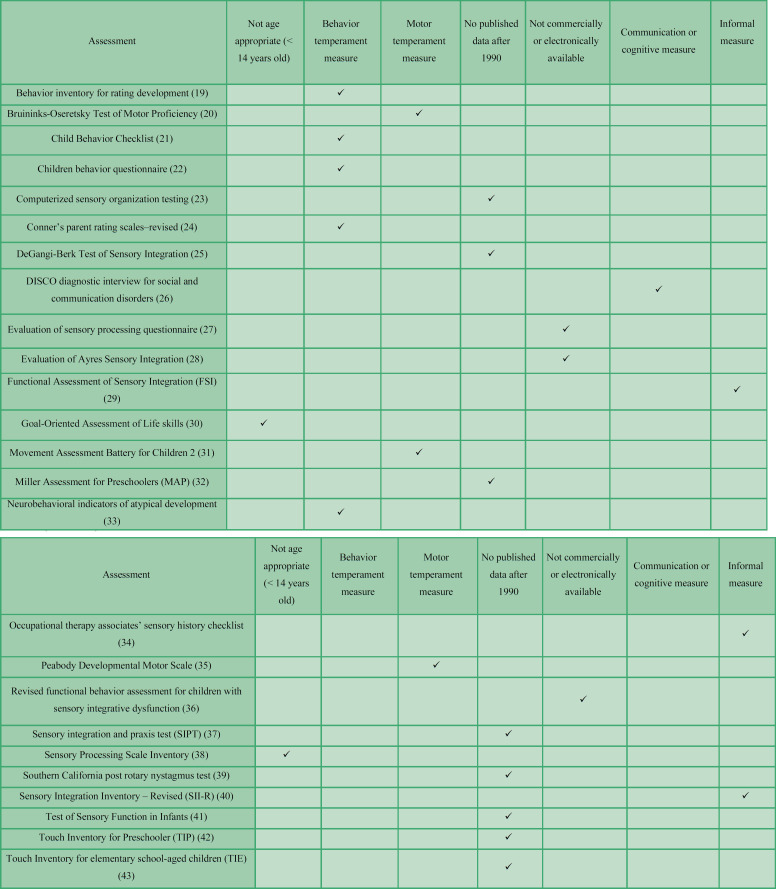

**Table 3:** Characteristics of included assessments

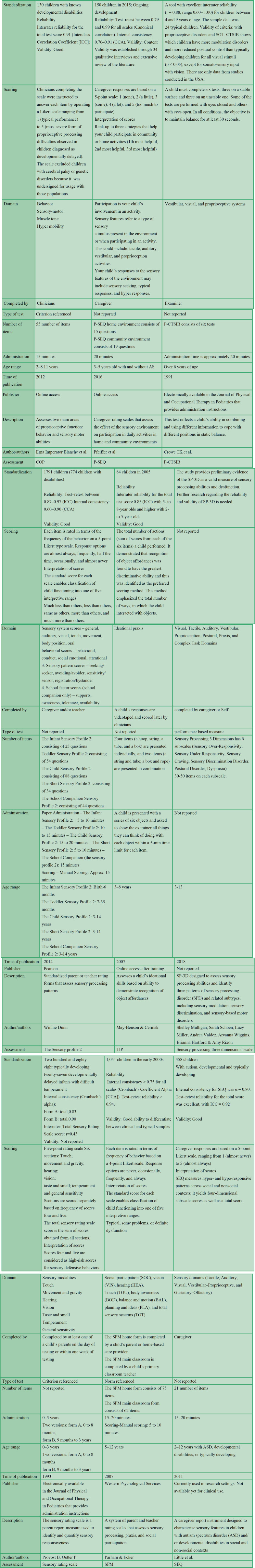

## Discussion

To the best of our knowledge, this is the first systematic review of valuable tools evaluating sensory processing in children within 0 and 14 years of age. This investigation may be of use to professionals to apply a suitable and valid sensory processing assessment tool for identifying and optimizing sensory processing in SPD patients.

The result of our research differs from those obtained in a systematic review administered by Eeles et al. These authors conducted a review to identify instruments available for measuring sensory processing in children aged 0 to 2 years (44). However, we carried out this systematic review to investigate sensory processing assessment tools designed for the 0-14 age group. In addition, our review differs from a study conducted by Jorquera-Cabrera et al. in terms of age range, search strategy, and inclusion criteria (45).

This study aimed to provide a comprehensive list of pediatric assessment tools particularly designed for sensory processing in children between 0 and 14 years of age. In this systematic review, nine sensory processing assessment tools in children aged between 0 and 14 years were identified from 1990 to 2019. P-CTSIB was the oldest tool, and the most recent tool was the sensory processing three dimensions’ scale, which were developed in 1993 and 2018, respectively. The maximum number of items was 243 in the sensory profile 2, and the minimum number of test items was 6 in TIP and P-CTSIB. According to these tests, the minimum and maximum age for performing the sensory profile 2 is 0 and 14 years, respectively. The sensory rating scale, SPM, SEQ; the sensory profile 2; P-SEQ and sensory processing 3 dimensions’ scale are the caregiver or teacher reported questionnaires. TIP, COP, and P-CTSIB are clinical observational assessment tools. The minimum testing time was 5 to 10 minutes for the infant sensory profile 2 and the maximum testing time was 20 minutes for P-SEQ. 

There are many tools for evaluating sensory processing in the first 14 years of life; nevertheless, we recommend professionals, particularly occupational therapists, to use the sensory profile 2. Reasons for using this tool are as follows:

- It has a broad age range (birth to 14:11).
- It has various administration options (paper and pencil or online through Q-global™).
- It includes a set of separate questionnaires related to age and various contexts (the infant, toddler, child, short, and school sensory profile 2).
- It considers broad domains (sensory system, behavioral pattern, sensory pattern, and school factors).
- Among the tools reviewed in this study, the highest sample size (1791 typical and atypical children) was used in the psychometric study of the test.
- It identifies behaviors that children exhibit as sensory processing patterns. It is based on a conceptual structure that proposes an interaction between neurological thresholds and self-regulatory behavioral responses, initially described by Dunn (1997).
- It provides a way to capture a child’s responses to sensory evidence during the course of routine life because each item describes an experience. Knowing how a child reacts in various contexts (home, school, and community) provides a way to comprehend what influences a child’s behavior throughout a day. All professionals must keep a primary focus on a child’s functional performance in ordinary life. To this end, the sensory profile 2 is a viable option because few evaluation tools measure performance in ordinary life in a specific context.
- Teachers and care providers reported therapeutic benefits after completing the sensory profile 2. Items in each rater questionnaire address activities and behaviors of infants, toddlers, and children common in most classroom settings. Responding to items about familiar behaviors provides validation that caregivers’ or teachers’ observations are relevant and offers opportunities to further discuss challenging situations.
- It is constructed so that families and professionals can engage in theory-based decision making during comprehensive assessment and intervention planning. Principles of neuroscience, sensory processing, strength-based approaches, and ecological models are embedded in its items and scoring system.
- It provides a standardized means to capture a child’s behaviors during the course of ordinary life, which is a challenging task to accomplish using other formal assessments conducted in unfamiliar settings. Prior work has illustrated that caregivers and teachers provide contextually relevant information about their own experiences to children, expanding our understanding of the impact of sensory processing on the demands of ordinary life.
- It provides a way to have a comprehensive look at a child’s responses across settings. Teachers and caregivers provide unique perspectives of a child’s performance because they interact with children in places and activities with various demands and supports. This facilitates discussion and collaboration among families and professionals to discover strategies that support a child’s participation in all contexts including home, school, and the community. Every so often a procedure works at home that can be used at school and vice versa; gathering all information together facilitates the discovery of effective strategies already in place.
- It presents a measure of current performance, overall impression over time, and an indication of intervention options. Test results provide information about a child’s level of responsivity to sensory events (e.g., hyper or hypo responsive). Since the sensory profile 2 is organized into sensory sections, test results also suggest which sensory systems might be supporting or interfering with a child’s performance in various settings and activities. Information gained from the sensory profile 2 provides a status measurement of current performance levels, and its scoring system provides guideposts for developing interventions (46).

## Conclusion

The strength of this review was that it presented a thorough and systematic search of relevant articles. To make this review more systematic and objective, the authors used standardized assessment structures to assess each study and examined the psychometric characteristic of the structures. As the limitation of the review, the authors did not include other suitable tools that are likely to be subjected to rigorous but unreported testing and thus have remained unpublished.
